# Universal Pharmacare – Redressing Social Inequities in the Canadian Health System: A Response to Recent Commentaries

**DOI:** 10.34172/ijhpm.2020.129

**Published:** 2020-07-18

**Authors:** Mohammad Hajizadeh, Sterling Edmonds

**Affiliations:** ^1^School of Health Administration, Dalhousie University, Halifax, NS, Canada.; ^2^Schulich School of Law, Dalhousie University, Halifax, NS, Canada.

We are grateful for the very interesting and insightful commentaries written by Lexchin,^1^ Tuohy,^2^ Acri née Lybecker,^3^ Rawson,^4^ and Gagnon^5^ in response to our editorial, “*Universal Pharmacare in Canada: A Prescription for Equity in Healthcare.”*^6^

In his commentary, Lexchin^1^ rightly highlights the numerous advantages to universal pharmacare in Canada, and that the main arguments against universal pharmacare (eg, pharmacare would be too costly, private insurance coverage would suffer, or only certain gaps in insurance coverage need filling) do not outweigh the strong arguments in favour of the regime. Tuohy^2^ offers a comprehensive commentary on the politics associated with pharmacare in Canada, and the similarities and differences with comparable debate in the United States and ‘Medicare for all.’ Likewise, Acri née Lybecker,^3^ drawing on the experience of the United States, makes the case that some Canadians (particularly lower-income Canadians, seniors, the disabled, and patients with chronic disease) currently have the coverage sought under a universal pharmacare program and that other options are worth exploring. Rawson^4^ discusses the importance of equity in any universal pharmacare regime, and that price of drugs will play an important role in their affordability and accessibility to the Canadian public. Finally, Gagnon^5^ correctly highlights the inequities (unfair inequalities) and inefficiencies found in the current system and that universal pharmacare would provide better access to prescription medications, instead of simply “filling the gap” with catastrophic coverage.

We agree with Acri née Lybecker that “the devil is in the detail” and we do not discount the complexity of implementing a single-payer, universal pharmacare program in Canada. The author asserts that, although many low-income Canadians may be struggling to pay for prescription drugs, these same low-income Canadians already have access to some form of provincial drug insurance limiting their out-of-pocket payments and that any social inequity would not be addressed by universal pharmacare anyway. We acknowledged and discussed in our editorial that significant interprovincial variation in public drug coverage has, in fact, led to corresponding variation in the burden of out-of-pocket expenses for drugs and pharmaceutical products.^6–8^ Residents should not have better or worse public drug coverage based solely on their province of residence, and universal pharmacare would fix that. Additionally, it should be restated that the social inequities highlighted in our editorial exist *despite* the existence of any provincial public drug coverage. In fact, catastrophic out-of-pocket expenses on drugs and pharmaceutical products disproportionately affect low-income households, rural households,^6^ seniors and households using social assistance.^7,9,10^ These inequities extend to those households that fall outside of the program criteria used by provincial governments to define, for instance, what a low-income household is. Our recent work on out-of-pocket payments for health care^11^ also indicated that many Canadians, including those who may not be poor, elderly, or using social assistance, face considerable inequities to finance their health care expenditures mainly due to the high out-of-pocket costs associated with drugs and pharmaceutical products.^11^ The reality is that the current system does not eliminate the risk of catastrophic payments on drugs and pharmaceutical products, and universal pharmacare could reduce this risk for Canadian households.

Both Acri née Lybecker and Rawson make the case that Canada may be better off expanding private insurance for the gaps in coverage that currently exist because they offer more generous coverage and coverage for more expensive drugs that would be left out of any public plans. This view is misleading because it misunderstands the reality of drug coverage, pricing, and access to prescription medicines in Canada.^5^ Gagnon rightly highlights that most private drug plans cover many new, increasingly expensive drugs that offer no significant therapeutic benefit, creating wasteful coverage regimes that do nothing to address the inequity of access among Canadians.^5,12^ As Lexchin points out, private plans that cover more drugs are not necessarily better than those plans that cover less,^1^ which is another potential source of wasteful coverage.

Several studies,^7,9,10^ including our recent editorial,^6^ suggest considerable social inequities in the burden of out-of-pocket expenses for drugs and pharmaceutical products in Canada. Patchwork public coverage with varying co-payments, deductibles, and provincial formularies burden so many Canadians, and universal pharmacare can potentially redress systemic social inequities that affect Canadian households.

## Ethical issues

Not applicable.

## Competing interests

Authors declare that they have no competing interests.

## Authors’ contributions

Both authors contributed to the conception, drafting, and revising of the correspondence.

## Authors’ affiliations

^1^School of Health Administration, Dalhousie University, Halifax, NS, Canada. ^2^Schulich School of Law, Dalhousie University, Halifax, NS, Canada.
